# Experience of annual events in the family and social adjustment of school-age children

**DOI:** 10.1186/s13034-022-00475-w

**Published:** 2022-06-03

**Authors:** Rikuya Hosokawa, Toshiki Katsura, Kazuya Taira

**Affiliations:** 1 Department of Human Health Sciences, Graduate School of Medicine, Kyoto University, Kyoto, 606-8507 Japan; 2 Faculty of Nursing Science, Meiji University of Integrative Medicine, Kyoto, 629-0392 Japan

**Keywords:** Annual event, Family experience, Cultural activities, Social celebrations, School age, Social adjustment, Prosocial behavior, Externalizing problem, Internalizing problem

## Abstract

**Background:**

Parent–child relationships, the rearing attitudes of parents toward their children as well as the interactive relationships, such as play and cultural activities that parents and children enjoy together, serve as important factors in predicting a child’s growth and development. These experiences of annual events celebrated with the family may be related to the school-age child’s development. However, this relationship has not been investigated sufficiently. Therefore, this study aimed to identify the relationship between the experience of annual events observed in the family and a child’s social adjustment.

**Methods:**

In 2019, a self-administered questionnaire survey targeting fifth graders (ages 10–11) in Japan was conducted with children’s parents. Major survey items included participants’ characteristics (child’s sex, family composition, siblings, household income, and parents’ educational backgrounds), annual events observed in the family (*Setsubun* or the day before the start of spring, Mother’s Day, Father’s Day, the *Tanabata* or Weaver Festival, Respect for the Aged Day, Winter solstice, etc.), and the child’s social adjustment (Strengths and Difficulties Questionnaire). A total of 653 children who met the criteria of not having any developmental disorders were included as participants for the analysis.

**Results:**

The participants had celebrated an average of 15.47 (± 5.52) annual events with their families that year. The number of annual events celebrated was significantly related to family composition and the parents’ educational backgrounds. We found that children who came from families with numerous experiences of annual events were more likely to have higher prosocial behavior and were less likely to have externalizing or internalizing problems. The same pattern was found even after adjusting for the family’s socioeconomic background and other factors; that is, children who came from families having diverse experiences of annual events were more likely to show prosocial tendencies.

**Conclusions:**

Our findings suggest that the experience of annual events observed with family potentially enhances a child’s prosocial behavior. Thus, celebrating and preserving cultural and personal events in the amily context may be an important developmental experience in terms of children’s social adjustment.

**Supplementary information:**

The online version contains supplementary material available at 10.1186/s13034-022-00475-w.

## Background

Researchers have shown that diverse experiences in daily life affect a child’s socio-emotional development [1]. Family experiences are important factors in predicting a child’s growth and development [2–4]. Family interactions that children experience include aspects of parent–child relationships, such as a parent’s warmth toward their child as well as sensitivity and responsiveness, and environmental aspects such as family resources and teaching materials [5]. Particularly in parent–child relationships, not only the rearing attitudes of parents for their children but also interactive relationships, such as play and cultural activities that both parents and children enjoy together, are important factors [5–8]. Family rituals include play and cultural activities that parents and children enjoy together. These rituals offer the opportunity to participate fully in family health [9–11]. Annual events represented by family rituals are special events such as celebrations, traditions, and patterned family interactions that have symbolic meanings shared by the entire family [10]. Family rituals were found to be related to increased parental competence, lower levels of anxiety, and marital satisfaction [9–11]. Moreover, they were also found to be related to medical adherence, mealtime interactions, and nighttime waking in children [9–11]. However, while the evidence of links between family rituals and family functioning is clear, the relationship between family rituals and child outcomes lacks sufficient clarity.

Countries worldwide have traditional family rituals as annual events that have been transmitted across generations for a long time and continue to be passed on as events unique to that particular country. Japan has a variety of traditional annual events that are held for each season throughout the year; there are also many annual events that, over time, have been adopted from other countries. Cultural experiences play an important role in fostering a child’s identity and building self-esteem [12]. Researchers have noted the importance of annual events conducted at educational sites during early childhood and elementary school years. The same has also been stated in the *Educational guidelines for kindergartens, nursery school childcare, elementary schools*, and in other documents [13–15]. The guidelines suggest that annual events are important for maintaining and promoting children’s health. Participating in annual events is a special experience for a child, and it deepens the cultural ties between the child and their family. Cultural experiences that have been fostered continue to be a part of the child’s life, even after they leave their parents’ home. There is a possibility that these experiences of annual events celebrated with the family are related to the child’s development. However, this relationship has not been sufficiently investigated. Therefore, this study aimed to identify the relationship between the experience of annual events observed in the family and the child’s social adjustment, using school-age children’s parents as participants. As indicated from prior evidence, annual events could promote prosocial behavior, better socio-emotional adjustment, and overall well-being in children, which makes it imperative to study this impact in greater detail to advance best practices in child development. Thus, findings of this study could contribute to interventions tailored to increase annual events in a child’s life to foster healthy development. The following research question was investigated in our study “how is the experience of annual family events related to children’s social adjustment.” Further, we hypothesize that diverse experiences of annual family events positively influence children’s social adjustment.

## Methods

This study was part of a research effort to examine the effects of parenting environment on children’s social development and adjustment, and was conducted in affiliation with Kyoto University. In 2014, parents of 5–6-year-old preschoolers were recruited as participants from 52 kindergartens and 78 nursery schools in Aichi Prefecture, a major metropolitan area in Japan. We followed up with these participants annually. Data for the current study was collected in 2019, from the same cohort of participants from 2014, during one of our annual follow-ups. In 2019, this cohort of children was aged 10–11-years and were in the fifth grade of elementary school. The parents (N = 1414) responded to self-reported paper-based questionnaires. The survey items included participants’ characteristics (child’s sex, family composition, siblings, household income, and parents’ educational backgrounds), description of the experience of annual events celebrated with the family during one year, and the child’s level of social adjustment. The questionnaires were distributed by mail to 1414 respondents and were returned by 720 of them. The total number of valid responses obtained was 709, after excluding 11 respondents with missing answers on items concerning child’s social adjustment, annual events, etc., which were necessary for this study. From the 709 valid responses, we excluded children with developmental disorders (n = 56) and used data for the remaining 653 children for our target analysis. We excluded data of children with developmental disabilities from this analysis because of their potential impact on annual family events performed in the family. For example, in the case of severe developmental disabilities, families may be reluctant to engage in certain annual family events. Thus, the addition of this cohort may have led to a high variance in responses as well as a misrepresentation of the impact of annual family events. Determination of developmental disability was based on parental report.

### Objective variable: child’s social adjustment

In this study, the children’s parents completed the Strengths and Difficulties Questionnaire (SDQ) (see Additional file 1: Appendix 1), which is a screening scale that is formulated to assess a child’s social adjustment using a paper-based questionnaire [16–18]. The SDQ is a 25-item measure of parents’ perceptions of their children’s prosocial and difficult behaviors, and is designed to assess internalizing and externalizing emotional and behavioral problems. The scale’s internal consistency and construct validity have been reported as adequate [19, 20]. The Japanese version of the SDQ was used in this study [21]. The participants responded to the questions using a 3-point Likert scale ranging from “not true” (0) to “certainly true” (2). It is composed of five subscales, namely, prosocial behavior (five items; e.g., considerate of other people’s feelings), conduct problems (five items; e.g., often has temper tantrums or hot tempers), hyperactivity/inattention (five items; e.g., restless, overactive, cannot stay still for long), peer relationship problems (five items; e.g., rather solitary, tends to play alone), and emotional problems (five items; e.g., often complains of headaches, stomach-aches or sickness). In this study, prosocial behaviors were designated as “prosocial behaviors,” conduct problems and hyperactivity/inattention were integrated into “externalizing problems,” and peer relationship problems and emotional problems were integrated into “internalizing problems.” Higher prosocial behavior scores indicated a greater degree of positive social behaviors, and higher externalizing and internalizing problem scores signified a greater degree of behavioral difficulties.

### Explanatory variable: annual events

The children’s parents also answered questions about the annual events observed in their family throughout the year, in a questionnaire-based survey (see Additional file 2: Appendix 2); the answers were close ended (yes/no). Specifically, they were asked about Japan’s major traditional annual events, such as New Year’s Day, *Nanakusa*—the seventh day of the New Year for eating the seven spring plants and vegetables, *Kagami-biraki—*cutting the New Year rice cake and eating it with a clear soup or as a sweet red bean soup, etc., *Setsubun*—throwing dry beans to expel demons, eating Ehomaki rolled sushi, etc. on the day before the start of spring, Doll’s Festival—displaying *Hina* dolls, eating special rice crackers and tossed sushi, etc., Children’s Day—displaying carp streamers, Boys’ Festival dolls, eating the event’s special sweets, etc., *Tanabata*—Weaver Festival, Bon Festival for remembering the dead, Respect for the Aged Day, Winter solstice, and New Year’s Eve. In terms of internationally popular annual events, they were asked about Valentine’s Day, Mother’s Day, Father’s Day, Halloween, Christmas, etc. They were also asked them about their family’s anniversaries, and children’s, parents’, and grandparent’s’ birthdays. In total, 28 kinds of annual events were included.

### Moderator variable: participants’ characteristics

In addition, the children’s parents provided data regarding the child’s sex, family composition, siblings, annual household income, and the parents’ educational backgrounds using a paper-based questionnaire. In this analysis, we established the child’s sex as boy or girl; the family’s composition as single-parent household or two-parent household; the family’s annual household income as less than three million yen, 3–6 million yen, 6–9 million yen, or more than nine million yen (1 yen is equivalent to about 0.0086 US dollars as of January 2022); and the parents’ educational backgrounds (or the highest educational level they have reached) as middle school or high school, junior college or vocational school, or university and graduate school.

### Statistical analysis

To investigate the relationships between the participants’ characteristics (child’s sex, family composition, siblings, annual household income, and parents’ educational backgrounds) and the number of annual events, as well as the relationship between the participants’ characteristics and social adjustment, we conducted analyses using the *t*-test and one-way analysis of variance.

Next, to investigate the relationship between the experience of annual events and the child’s social adjustment, we considered social adjustment (prosocial behavior, externalizing problems, and internalizing problems) as the objective variable and the number of annual events as the explanatory variable. We then conducted a multiple regression analysis. In Model l, analysis was performed without including moderator variables, and in Model 2, it was performed by establishing the following as the moderator variables: child’s sex, family composition, siblings, annual household income, and parents’ educational backgrounds.

## Results

### Participants’ characteristics and annual events

Table 1 shows the participants’ characteristics, whereas Table 2 shows the description of annual events. The number of annual events observed in families was 15.47 (± 5.52).

**Table 1:** Participants’ characteristics

	n	%
Child’s sex		
Boy	314	48.1
Girl	339	51.9
Family composition		
Single-parent household	44	6.7
Two-parent household	609	93.3
Siblings		
No	107	16.4
Yes	546	83.6
Annual household income		
Less than 3 million yen	56	8.6
3–6 million yen	268	41.0
6–9 million yen	184	28.2
9 million yen or more	131	20.1
Mother’s educational background		
Middle school or high school	140	21.4
Junior college or vocational school	265	40.6
University or graduate school	241	36.9
Father’s educational background		
Middle school or high school	166	25.4
Junior college or vocational school	90	13.8
University or graduate school	370	56.7
Overall	653	100.0

**Table 2:** List of annual events

Period	Content	%
Jan	New Year (making the first visit of the year to the shrine; eating traditional New Year dishes, etc.)	96.0
Jan	*Nanakusa* (eating soup containing seven spring plants and vegetables, on the seventh day of the New Year)	30.8
Jan	*Kagami-biraki* (cracking a huge rice cake and eating it with a clear soup or as a sweet red bean soup, etc.)	41.7
Feb	*Setsubun* (throwing dry beans to expel demons, eating *Ehomaki* rolled sushi, etc.)	81.5
Feb	Valentine’s Day (giving and/or receiving chocolates, etc.)	84.1
March	Doll’s Festival (displaying *Hina* dolls, eating special rice crackers and tossed *sushi*, etc.)	70.6
March	White Day (giving and/or receiving presents, etc.)	51.0
March	The spring Equinoctial week (visiting the family grave, eating special rice cakes, etc.)	24.0
March–April	Viewing cherry blossoms (enjoy looking at cherry and plum blossoms; eating rice cakes rolled in pickled cherry leaf)	67.1
April	Easter (eating egg dishes and special feasts, etc.)	4.7
May	Children’s Day (displaying carp streamers and Boys’ Festival dolls, eating the day’s special sweets, etc.)	73.2
May	Mother’s Day (giving a gift to one’s mother, etc.)	62.6
June	Father’s Day (giving a gift to one’s father, etc.)	59.9
July	*Tanabata* (Weaver’s Festival, putting up bamboo decorations, etc.)	35.8
July-Aug	The Midsummer Day of the Ox (eating grilled eel, etc.)	35.1
Aug	Bon Festival (visiting the family grave, etc.)	69.5
Sept	The autumn Equinoctial week (visiting the family grave, eating special rice cakes, etc.)	26.5
Sept	Respect for the Aged Day (giving gifts to grandparents, etc.)	38.6
Sept–Oct	Moon viewing (looking at the moon on the 13th and 15th nights of the lunar month; eating special dumplings, etc.)	37.2
Oct	Halloween (dressing up, making decorations, etc.)	50.4
Nov–Dec	Viewing of autumn leaves (enjoy looking at leaves that have turned red and yellow, etc.)	30.6
Dec	Winter solstice (taking a hot bath with dried citrus peel floating in it, eating pumpkin dishes, etc.)	45.8
Dec	Christmas (decorating a Christmas tree, eating Christmas cake, etc.)	96.8
Dec	New Year’s Eve (eating traditional *soba* noodles just before midnight, etc.)	92.3
–	Children’s birthday	98.8
–	Parents’ birthdays	75.5
–	Grandparents’ birthdays	45.9
–	Parents’ wedding anniversary	18.5

### Number of annual events by participants’ characteristics

Table 3 shows the number of annual events by participants’ characteristics. Girls reported experiencing a greater number of events than boys. Two-parent households experienced a greater number of events than single-parent households. The higher the mother’s educational background, the greater the number of events the child experienced.

**Table 3:** Number of annual events by participants’ characteristics

	M	SD	P-value
Child’s sex			
Boy	14.47	5.61	< 0.001
Girl	16.41	5.27	
Family composition			
Single-parent household	12.68	5.24	< 0.001
Two-parent household	15.68	5.49	
Siblings			
No	14.56	6.28	0.094
Yes	15.65	5.35	
Annual household income			
Less than 3 million yen	13.79	5.60	0.124
3–6 million yen	15.68	5.50	
6–9 million yen	15.58	5.10	
More than 9 million yen	15.27	5.93	
Mother’s educational background			
Middle school or high school	14.34	5.89	0.011
Junior college or vocational school	15.48	5.28	
University or graduate school	16.09	5.45	
Father’s educational background			
Middle school or high school	15.16	5.63	0.621
Junior college or vocational school	15.67	5.81	
University or graduate school	15.64	5.38	
Overall	15.47	5.52	

### Participants’ characteristics and the child’s social adjustment

Table 4 shows the participants’ characteristics and the child’s social adjustment. By sex, boys had lower scores for prosocial behaviors and higher scores for externalizing problems than girls. Regarding family composition, single-parent households had higher internalizing problems scores than two-parent households. In terms of the presence or absence of siblings, those who had no siblings had higher internalizing problems scores than those who had siblings. The lower the household income and mother’s educational background, the higher the child’s internalizing problems score.

**Table 4:** Participants’ characteristics and the child’s social adjustment behaviors

	Prosocial behaviors			Externalizing problems			Internalizing problems		
	M	SD	P-value	M	SD	P-value	M	SD	P-value
Child’s sex									
Boy	6.27	2.22	< 0.001	5.26	3.21	< 0.001	3.50	3.17	0.575
Girl	7.07	2.05		3.97	2.92		3.37	2.80	
Family composition									
Single-parent household	6.50	2.52	0.571	4.79	3.24	0.671	4.58	3.30	0.013
Two-parent household	6.70	2.14		4.57	3.12		3.36	2.95	
Siblings									
No	6.50	2.09	0.319	4.79	3.30	0.481	4.01	3.28	0.033
Yes	6.73	2.18		4.55	3.10		3.33	2.92	
Annual household income									
Less than 3 million yen	6.36	2.07	0.133	4.83	3.17	0.282	4.42	3.62	0.024
3–6 million yen	6.90	2.17		4.73	3.39		3.56	3.00	
6–9 million yen	6.47	2.21		4.69	3.09		3.42	2.93	
More than 9 million yen	6.62	2.11		4.13	2.55		2.95	2.76	
Mother’s educational background									
Middle school or high school	6.53	1.93	0.068	4.92	3.45	0.367	4.06	2.96	0.021
Junior college or vocational school	6.91	2.16		4.55	3.08		3.24	3.21	
University or graduate school	6.50	2.27		4.45	2.98		3.27	2.65	
Father’s educational background									
Middle school or high school	6.92	2.10	0.233	4.80	3.11	0.209	3.83	3.30	0.122
Junior college or vocational school	6.70	2.14		4.92	3.55		3.20	2.90	
University or graduate school	6.57	2.19		4.39	3.05		3.30	2.85	
Overall	6.69	2.17		4.59	3.13		3.44	2.99	

### Relationship between number of annual events observed in the family and the child’s social adjustment

Table 5 shows the relationship between number of annual events celebrated in the family and the child’s prosocial behavior. Table 6 shows the relationship between number of annual events celebrated in the family and the child’s externalizing problems, whereas Table 7 shows the relationship between number of annual events celebrated in the family and the child’s internalizing problems. In Model 1, which had no moderating variables input, the greater the number of annual events, the higher the child’s prosocial behavior and the lower their externalizing and internalizing problems. In Model 2, which included the child’s sex, family composition, siblings, annual household income, and the parents’ educational backgrounds as moderating variables, the same pattern was observed: the greater the number of annual events, the higher the child’s prosocial behavior.

**Table 5:** Relationship between number of annual events celebrated in the family and the child’s prosocial behavior

	Model 1					Model 2				
	B	SE	β	P-value	Adjusted R^2^	B	SE	β	P-value	Adjusted R^2^
Number of annual events	0.069	0.015	0.177	< 0.001	0.030	0.055	0.016	0.141	< 0.001	0.050
(Moderator variables)										
Child’s sex	–	–	–	–	–	0.740	0.176	0.172	< 0.001	
Family composition	–	–	–	–	–	0.134	0.488	0.011	0.784	
Siblings	–	–	–	–	–	0.069	0.240	0.012	0.773	
Annual household income	–	–	–	–	–	− 0.075	0.105	− 0.031	0.475	
Mother’s educational background	–	–	–	–	–	− 0.069	0.128	− 0.024	0.586	
Father’s educational background	–	–	–	–	–	− 0.139	0.109	− 0.056	0.204	

Analysis method: multiple regression analysis. Model 1: Input independent variables. Model 2: Input all the independent variables and moderator variables (child’s sex, family composition, siblings, household income, and parents’ educational backgrounds)

**Table 6:** Relationship between number of annual events celebrated in the family and the child’s externalizing problems

	Model 1					Model 2				
	B	SE	β	P-value	Adjusted R^2^	B	SE	β	P-value	Adjusted R^2^
Number of annual events	− 0.062	0.022	− 0.110	0.006	0.011	− 0.037	0.024	− 0.065	0.120	0.033
(Moderator variables)										
Child’s sex	–	–	–	–	–	− 1.094	0.258	− 0.175	< 0.001	
Family composition	–	–	–	–	–	− 0.071	0.683	− 0.004	0.918	
Siblings	–	–	–	–	–	− 0.059	0.356	− 0.007	0.868	
Annual household income	–	–	–	–	–	− 0.135	0.155	− 0.038	0.383	
Mother’s educational background	–	–	–	–	–	0.012	0.188	0.003	0.951	
Father’s educational background	–	–	–	–	–	− 0.160	0.161	− 0.044	0.320	

Analysis method: multiple regression analysis. Model 1: Input independent variables. Model 2: Input all the independent variables and moderator variables (child’s sex, family composition, siblings, household income, and parents’ educational backgrounds).

**Table 7:** Relationship between number of annual events celebrated in the family and the child’s internalizing problems

	Model 1					Model 2				
	B	SE	β	P-value	Adjusted R^2^	B	SE	β	P-value	Adjusted R^2^
Number of annual events	− 0.051	0.021	− 0.094	0.017	0.007	− 0.032	0.023	− 0.058	0.164	0.015
(Moderator variables)										
Child’s sex	–	–	–	–	–	0.009	0.248	0.002	0.971	
Family composition	–	–	–	–	–	− 1.059	0.673	− 0.065	0.116	
Siblings	–	–	–	–	–	− 0.483	0.341	− 0.058	0.158	
Annual household income	–	–	–	–	–	− 0.241	0.149	− 0.071	0.105	
Mother’s educational background	–	–	–	–	–	− 0.161	0.181	− 0.040	0.375	
Father’s educational background	–	–	–	–	–	− 0.095	0.154	− 0.028	0.538	

Analysis method: multiple regression analysis. Model 1: Input independent variables. Model 2: Input all the independent variables and moderator variables (child’s sex, family composition, siblings, household income, and parents’ educational backgrounds)

## Discussion

In this study, the number of annual events experienced was significantly related to family composition and the parents’ educational backgrounds. Single-parent households experienced fewer events than two-parent households. The lower the mother’s educational background, the fewer the events experienced by the child. Studies have found that families with a low socio-economic status (SES) tended to have a low-quality family environment [5, 22]. The possible explanation for this includes lack of economic resources, differences due to the parents’ educational backgrounds, and mental factors such as stress caused by poverty. Our findings suggest the possibility that celebrating fewer annual events in a family is related to low SES. Prior research has shown that family rituals are related to children’s academic achievement [10, 23]. This suggests that an intergenerational cycle of negative outcomes may be occurring through family rituals; the lower the parents’ educational background, the fewer the family rituals, and consequently, the lower the children’s academic achievement. Concurrently, family rituals may be a factor that prevents negative intergenerational patterns.

In this study, we investigated the relationship between the experience of annual events and the child’s social adjustment. We found that children from families with numerous annual events were more likely to show higher prosocial behavior and that their externalizing/internalizing problems were likely to be less acute. Even after adjusting for families’ socioeconomic background and other factors, the trend was similar. Although the Adjusted R^2^ was not high, it was meaningful as it indicated a significant association between family experiences and behavior in children. Annual events represented by family rituals are special events such as celebrations, traditions, and patterned family interactions that have symbolic meanings shared by the entire family [10]. Family rituals are uniquely meaningful family interactions that communicate family beliefs and values and provide families with a sense of stability, identity, and a means of socialization [24]. Previous studies have shown that family rituals enhance parent-child interaction and that the amount of parent-child interaction affects children’s language ability and development [25]. In addition, family rituals are associated with positive outcomes such as family quality of life, psychological functioning, and health-related behaviors [26, 27]. These findings suggest that family rituals may enhance children’s social adjustment. In Japan, with the continuing decline in birthrates and the rising ratio of nuclear families, the transmission of dietary culture between generations has slowed [28]. This may indicate the reduced transference of other cultural activities as well, and with communities undergoing such changes, it becomes even more crucial to actively propagate the transmission of traditional cultural events. This is especially important in the context of the findings of our study, that indicate positive social adjustment in children is directly linked to family rituals. Our findings indicate that family rituals may be an important factor in fostering social adjustment in children.

### Study limitations

This study has several limitations. First, because this was a cross-sectional study, it was not possible to examine causal relationships. Second, although we investigated annual family events that are popular in Japan, several events were not included in the list. Third, we looked at limited aspects of children’s social adjustment using the SDQ. Fourth, out of a distribution of 1414 participants, this study had 709 valid responses, of which 653 were used for analysis, which may indicate bias in the selection of respondents. Finally, this study was conducted in a limited region in Japan, which may affect generalizability. We hope that future studies take these points into consideration for further research related to our findings.

## Conclusions

This study used a sample of school-age children to identify the relationship between children’s experiences of cultural and personal events celebrated with their families and their social adjustment. Our findings suggest that the experience of annual events is likely to enhance a child’s prosocial behavior. Previously, it has been observed that the diversity of a child’s experiences, both inside and outside the home, is affected by the family’s SES. Even after adjusting for background factors such as household income and parents’ educational backgrounds, the number of experiences was shown to relate positively to prosocial behavior. Thus, celebrating and preserving annual events with the entire family plays an important role in a child’s social adjustment across a variety of family environments. The results of this study will contribute to the literature on environmental factors in child-rearing that promote the development of children’s social abilities and social adjustment.

## Supplementary information

**Additional file 1: Appendix 1.** Strengths and Difficulties Questionnaire.

**Additional file 2: Appendix 2.** List of annual events.

## Data Availability

The datasets generated and/or analyzed during the current study are not publicly available, but are available from the corresponding author on reasonable request.

## Abbreviations

SDQ: Strengths and Difficulties Questionnaire.
SES: Socio-economic status.
