# Supplementary material for: Experience of annual events in the family and social adjustment of school-age children
Source: Child Adolesc Psychiatry Ment Health. 2022 Jun 3;16:39. doi: 10.1186/s13034-022-00475-w (PMC9166509; doi:10.1186/s13034-022-00475-w)
Supplement: Supplementary file 1 — Additional file 1: Appendix 1. Strengths and Difficulties Questionnaire. [file 13034_2022_475_MOESM1_ESM.docx]

| **Appendix 1** Strengths and Difficulties Questionnaire | |  |
| --- | --- | --- |
| Item | | Subscale |
| 1 | Considerate of other people's feelings | Prosocial behavior |
| 2 | Restless, overactive, cannot stay still for long | Hyperactivity/inattention |
| 3 | Often complains of headaches, stomach-aches or sickness | Emotional problems |
| 4 | Shares readily with other children (treats, toys, pencils etc.) | Prosocial behavior |
| 5 | Often has temper tantrums or hot tempers | Conduct problems |
| 6 | Rather solitary, tends to play alone | Peer relationship problems |
| 7 | Generally obedient, usually does what adults request | Conduct problems |
| 8 | Many worries, often seems worried | Emotional problems |
| 9 | Helpful if someone is hurt, upset or feeling ill | Prosocial behavior |
| 10 | Constantly fidgeting or squirming | Hyperactivity/inattention |
| 11 | Has at least one good friend | Peer relationship problems |
| 12 | Often fights with other children or bullies them | Conduct problems |
| 13 | Often unhappy, down-hearted or tearful | Emotional problems |
| 14 | Generally liked by other children | Peer relationship problems |
| 15 | Easily distracted, concentration wanders | Hyperactivity/inattention |
| 16 | Nervous or clingy in new situations, easily loses confidence | Emotional problems |
| 17 | Kind to younger children | Prosocial behavior |
| 18 | Often lies or cheats | Conduct problems |
| 19 | Picked on or bullied by other children | Peer relationship problems |
| 20 | Often volunteers to help others (parents, teachers, other children) | Prosocial behavior |
| 21 | Thinks things out before acting | Hyperactivity/inattention |
| 22 | Steals from home, school or elsewhere | Conduct problems |
| 23 | Gets on better with adults than with other children | Peer relationship problems |
| 24 | Many fears, easily scared | Emotional problems |
| 25 | Sees tasks through to the end, good attention span | Hyperactivity/inattention |
| Note: Goodman R. The Strengths and Difficulties Questionnaire: A research note. J Child Psychol Psychiatry. 1997;38:581–6. doi:10.1111/j.1469-7610.1997.tb01545.x | | |
