# Supplementary material for: Experience of annual events in the family and social adjustment of school-age children
Source: Child Adolesc Psychiatry Ment Health. 2022 Jun 3;16:39. doi: 10.1186/s13034-022-00475-w (PMC9166509; doi:10.1186/s13034-022-00475-w)
Supplement: Supplementary file 2 — Additional file 2: Appendix 2. List of annual events. [file 13034_2022_475_MOESM2_ESM.docx]

| **Appendix 2** List of annual events | |
| --- | --- |
| Period | Content |
| Jan | New Year (making the first visit of the year to the shrine; eating traditional New Year dishes, etc.) |
| Jan | *Nanakusa* (eating soup containing seven spring plants and vegetables, on the seventh day of the New Year) |
| Jan | *Kagami-biraki* (cracking a huge rice cake and eating it with a clear soup or as a sweet red bean soup, etc.) |
| Feb | *Setsubun* (throwing dry beans to expel demons, eating *Ehomaki* rolled sushi, etc.) |
| Feb | Valentine’s Day (giving and/or receiving chocolates, etc.) |
| March | Doll’s Festival (displaying *Hina* dolls, eating special rice crackers and tossed *sushi,* etc.) |
| March | White Day (giving and/or receiving presents, etc.) |
| March | The spring Equinoctial week (visiting the family grave, eating special rice cakes, etc.) |
| March–April | Viewing cherry blossoms (enjoy looking at cherry and plum blossoms; eating rice cakes rolled in pickled cherry leaf) |
| April | Easter (eating egg dishes and special feasts, etc.) |
| May | Children’s Day (displaying carp streamers and Boys’ Festival dolls, eating the day’s special sweets, etc.) |
| May | Mother’s Day (giving a gift to one’s mother, etc.) |
| June | Father’s Day (giving a gift to one’s father, etc.) |
| July | *Tanabata* (Weaver’s Festival, putting up bamboo decorations, etc.) |
| July-Aug | The Midsummer Day of the Ox (eating grilled eel, etc.) |
| Aug | Bon Festival (visiting the family grave, etc.) |
| Sept | The autumn Equinoctial week (visiting the family grave, eating special rice cakes, etc.) |
| Sept | Respect for the Aged Day (giving gifts to grandparents, etc.) |
| Sept–Oct | Moon viewing (looking at the moon on the 13th and 15th nights of the lunar month; eating special dumplings, etc.) |
| Oct | Halloween (dressing up, making decorations, etc.) |
| Nov–Dec | Viewing of autumn leaves (enjoy looking at leaves that have turned red and yellow, etc.) |
| Dec | Winter solstice (taking a hot bath with dried citrus peel floating in it, eating pumpkin dishes, etc.) |
| Dec | Christmas (decorating a Christmas tree, eating Christmas cake, etc.) |
| Dec | New Year’s Eve (eating traditional *soba* noodles just before midnight, etc.) |
| ― | Children’s birthday |
| ― | Parents’ birthdays |
| ― | Grandparents’ birthdays |
| ― | Parents’ wedding anniversary |
